# Association Between Growth Differentiation Factor-15 and Coagulation Parameters in Male Chinese Patients With Coronary Artery Disease

**DOI:** 10.31083/RCM43150

**Published:** 2026-01-09

**Authors:** Huan Liu, Yongnan Lyu, Wen Dai, Yan Li

**Affiliations:** ^1^Department of Clinical Laboratory, Renmin Hospital of Wuhan University, 430060 Wuhan, Hubei, China; ^2^Department of Cardiology, Renmin Hospital of Wuhan University, 430060 Wuhan, Hubei, China

**Keywords:** growth differentiation factor-15, coronary artery disease, coagulation, acute coronary syndrome

## Abstract

**Background:**

Growth differentiation factor-15 (GDF-15) has emerged as a novel biomarker for coronary artery disease (CAD). Although the hypercoagulable state is recognized as a biological mechanism that triggers cardiac events in CAD, the relationship between GDF-15 and coagulation parameters in patients with CAD remains unclear. Thus, this study aimed to investigate the potential relationship between GDF-15 and coagulation parameters in male Chinese patients with CAD.

**Methods:**

In total, 892 subjects were enrolled between January 2020 and December 2020, including 592 with CAD and 300 controls. The serum levels of GDF-15, blood cell count, glucose, serum lipids, and coagulation parameters were measured. Kruskal–Wallis or one-way ANOVA with post hoc tests (Holm–Sidak and Dunn's tests), as well as univariate/multivariate linear regression analyses, were used to determine the correlation between GDF-15 and coagulation parameters in male patients with CAD.

**Results:**

Compared to controls, patients with acute myocardial infarction (AMI) and stable angina (SA) showed significantly higher levels of GDF-15 (*p* < 0.05). Multivariate linear regression revealed that GDF-15 levels were positively associated with activated partial thromboplastin time (APTT) in patients with CAD (β = 0.109, *p *= 0.024), and inversely associated with antithrombin III (AT3) (β = –0.113, *p *= 0.028) in an adjusted multivariate regression model. Meanwhile, in a multivariate regression model adjusted for other variables, the GDF-15 levels in patients with SA were inversely associated with AT3 (β = –0.191, *p *= 0.036). After adjusting for confounders, the GDF-15 levels were positively associated with APTT (β = 0.174, *p *= 0.002) and inversely associated with monocyte count (β = –0.159, *p *= 0.025) in patients with AMI.

**Conclusions:**

Elevated levels of GDF-15 in male CAD patients are associated with altered coagulation parameters, suggesting that GDF-15 may serve as a compensatory marker for coagulation parameter instability. These results underscore the potential clinical value of GDF-15 as a novel biomarker for assessing the coagulation status in patients with CAD, especially in the acute coronary syndrome (ACS) subgroup.

## 1. Introduction

Coronary artery disease (CAD), the leading cause of death worldwide, is characterized by reversible myocardial ischemia due to demand/supply mismatch [1]. Acute coronary syndromes (ACS), the primary clinical manifestation of coronary atherosclerosis, are typically caused by plaque rupture and subsequent thrombus formation in epicardial arteries, leading to acute occlusion [2]. Thrombosis plays a central role in the pathophysiology of ACS, which is driven by interrelated mechanisms, such as endothelial dysfunction, inflammation, and coagulation. Systemic inflammation promotes a proatherogenic state by upregulating prothrombotic factors and cell adhesion molecules, thereby activating platelets and facilitating clot formation [3]. Thus, coagulation status critically affects the development and progression of CAD.

Growth differentiation factor-15 (GDF-15), a member of the transforming growth factor-β superfamily [4, 5], is markedly upregulated in pathological conditions, like inflammation, cardiac injury, and oxidative stress [6, 7, 8]. Emerging evidence highlights the role of GDF-15 as a biomarker in cardiovascular diseases, including CAD [9, 10, 11] and links it to thrombosis. In early atherosclerosis, GDF-15 recruits macrophages to plaques [12]. Consistently, GDF-15 deficiency enhances plaque stability by reducing macrophage migration and increasing collagen deposition [13]. Notably, GDF-15 independently predicts bleeding risk in atrial fibrillation [14, 15, 16] and correlates with the risk of venous thromboembolism risk and thrombus burden in deep venous thrombosis [17, 18]. However, direct evidence linking GDF-15 to coagulation parameters in CAD remains scarce.

The prognostic value of GDF-15 in CAD, which predicts mortality and disease progression [9], makes it clinically relevant [19, 20]. Concurrently, assessing the coagulation status is vital for managing the risk of thrombosis/bleeding. Elevated levels of GDF-15 after ACS are associated with bleeding risk [15], and thrombogenesis in acute myocardial infarction (AMI) alters peripheral vascular responses compared to stable CAD [21]. GDF-15 combined with conventional coagulation markers, such as activated partial thromboplastin time (APTT), which correlates with the clinical presentation of patients undergoing angiography [22], may help guide the management of CAD. Although no studies have directly investigated the association between GDF-15 and coagulation in CAD.

This study is the first to investigate the associations between GDF-15 and coagulation parameters (prothrombin time, (PT), APTT, fibrinogen, thrombin time (TT), D-dimer, and antithrombin III (AT3)) in male patients with CAD, including those with ACS and SA subgroups.

## 2. Materials and Methods

### 2.1 Study Population and Eligibility Criteria

**Fig. 1.**
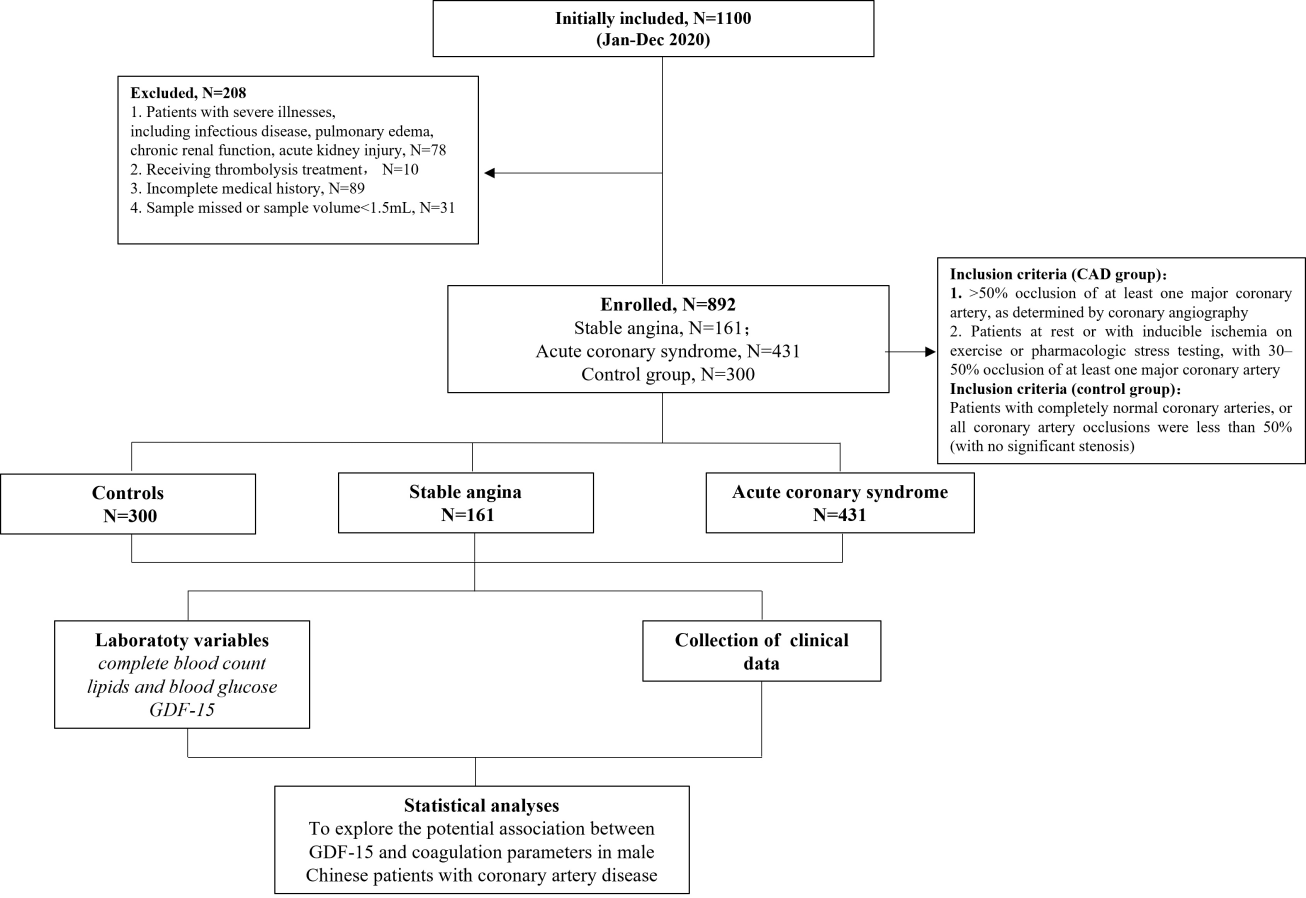
**Diagram of patient selection**. GDF-15, growth differentiation factor-15; CAD, coronary artery disease.

This retrospective cross-sectional study enrolled male patients who underwent elective coronary angiography for suspected CAD (including SA and ACS) at the Renmin Hospital of Wuhan University between January 2020 and December 2020. Individuals with other severe comorbidities, such as active infection, pulmonary edema, chronic/acute kidney injury, or those undergoing recent thrombolysis, were excluded. In total, 892 patients were finally enrolled. Participants with ≥50% stenosis in one major coronary artery or 30%–50% stenosis in coronary angiography with evidence of ischemia (resting or stress-induced) were included in the CAD group. Patients with completely normal coronary arteries and those with all coronary artery occlusions less than 50% without significant stenosis were included in the control group (Fig. 1).

The study protocol was approved by the Medical Ethics Review Committee of Renmin Hospital, Wuhan University, and complied with the Declaration of Helsinki. Written informed consent was obtained from all participants.

### 2.2 Angiography

Two blinded radiologists evaluated the severity of stenosis based on the Gensini score [23]. Vascular disease was classified as 0–3 based on the affected arteries (left anterior descending, left circumflex, right coronary). Left main trunk involvement was scored as 2-vessel disease.

### 2.3 Definitions

In this study, SA was defined as a myocardial ischemia/hypoxia due to ≥50% coronary occlusion [24]. ACS was defined as ST-segment elevation myocardial infarction, non-ST-segment elevation myocardial infarction, and unstable angina following the current guidelines [25].

### 2.4 Laboratory Measurements

Blood samples from patients with AMI were collected immediately upon hospitalization due to the urgency of their medical condition. For other patients, venipuncture was conducted in the morning after an overnight fast. Venous blood sample was drawn into plain tubes, centrifuged at 3500 rpm/min at 25 °C for 15 minutes, and the separated plasma/serum was stored at –80 °C until analysis.

Complete blood count (white blood count (WBC), neutrophils (Neu), monocytes (Mono), and lymphocytes (Lym)) was analyzed using a Sysmex XN-20 hematology analyzer (Kobe, Japan). Serum biochemical parameters (total cholesterol (TC), triglyceride (TG), high-density lipoprotein cholesterol (HDL-c), low-density lipoprotein cholesterol (LDL-c), and glucose) were quantified using an ADVIA 2400 biochemistry analyzer (Siemens, Munich, Germany). GDF-15 levels were measured via Elecsys® electrochemiluminescent immunoassay (Roche Diagnostics, Basel, Switzerland; research-use-only in China; detection range: 400–20,000 ng/L). Coagulation tests (APTT, PT, TT, and fibrinogen) were conducted on a CS-5100 analyzer (Sysmex, Kobe, Japan) using the clotting method, while D-dimer (immunoturbidimetry) and AT3 (chromogenic substrate method) were assayed on the same platform.

All procedures were conducted strictly following the specifications by the manufacturer and package insert guidelines.

### 2.5 Statistical Analysis

Continuous variables are presented as median (interquartile range [IQR]) or mean ± SD, and categorical variables are summarized as frequencies (percentages) for baseline characteristics and laboratory parameters. Patients with CAD were divided into high, medium, and low GDF-15 groups based on their tertiles [26]. Continuous variables were compared among the three groups using one-way ANOVA or the nonparametric Kruskal-Wallis test. For significant omnibus tests (*p* < 0.05), post hoc analyses were conducted using the following approaches: (a) Holm-Sidak method for normally distributed data, or (b) Dunn’s test with Holm adjustment for data with non-normal distributions. All comparisons were conducted at a 5% overall significance level. Categorical variables were analyzed using the Pearson chi-square test or Fisher’s exact test, as appropriate. Univariate/multivariate linear regression was employed to assess the relationship between serum levels of GDF-15 and coagulation parameters. Independent variables were selected through stepwise regression to avoid collinearity and to fit the optimal model.

A two-sided *p*-value < 0.05 was considered statistically significant. Analyses were conducted using IBM SPSS 23.0 (IBM Corp., Armonk, NY, USA) and GraphPad Prism 7.0 (GraphPad Software, La Jolla, CA, USA).

## 3. Results

### 3.1 Baseline Characteristics

There were no significant differences in terms of the distribution of age, hypertension, diabetes and smoking among patients with SA, those with ACS, and controls (*p* > 0.05) (Table 1). However, the levels of inflammatory markers (WBC, Neu, and Mono), glucose, and TG in the SA, and ACS groups were higher than those in the control group (*p* < 0.05). Additionally, the SA and ACS groups had significantly lower Lym and PLT count (*p* < 0.05). Notably, GDF-15 levels were significantly higher in the ACS group compared to the control and SA groups (*p* < 0.05).

**Table 1.** **Baseline characteristics of the study population**.

Characteristics	Controls (n = 300)	SA (n = 161)	ACS (n = 431)	*p*-value
Age, years	61.00 (56.00–67.0)	56.00 (51.00–62.00)	65.00 (58.00–71.00)	0.170
Diabetes, n (%)	69 (23.00)	40 (24.84)	117 (27.15)	0.271
Hypertension, n (%)	170 (56.67)	93 (57.76)	267 (61.95)	0.258
Smoking, n (%)	158 (52.67)	102 (63.35)	248 (57.54)	0.073
WBC (10^9^/L)	5.81 (5.05–6.66)	6.63 (5.65–7.43)	6.70 (5.39–8.38)	<0.001
Neu (10^9^/L)	3.25 (2.55–3.86)	4.01 (3.10–4.90)	4.12 (3.15–5.60)	<0.001
Lym (10^9^/L)	2.01 ± 0.67	1.71 ± 0.58	1.61 ± 0.64	<0.001
Mono (10^9^/L)	0.43 (0.37–0.53)	0.48 (0.41–0.63)	0.56 (0.44–0.69)	<0.001
PLT (10^9^/L)	221.96 ± 53.15	205.91 ± 63.23	205.14 ± 72.16	0.002
Glu (mmol/L)	4.51 (4.18–4.89)	4.90 (4.52–5.85)	5.20 (4.55–6.86)	<0.001
TC	4.39 (3.93–4.80)	3.84 (3.26–4.66)	3.67 (3.08–4.41)	<0.001
TG	1.11 ± 0.42	1.63 ± 0.86	1.63 ± 2.66	0.001
HDL-c	1.26 ± 0.24	0.98 ± 0.25	0.96 ± 0.29	<0.001
LDL-c	2.48 ± 0.51	2.15 ± 0.85	2.13 ± 0.86	0.562
GDF-15 (pg/mL)	700.00 (545.00–985.00)	775.00 (655.50–960.50)	1617.00 (1164.00–2286.00)	<0.001

Data are expressed as median (25th–75th percentile) or mean ± SD; nominal data are presented as percentages. Kruskal-Wallis test and one-way ANOVA were conducted for comparison. ACS, acute coronary syndromes; WBC, white blood count; Neu, neutrophils; Mono, monocytes; Lym, lymphocytes; TC, total cholesterol; TG, triglyceride; HDL-c, high-density lipoprotein cholesterol; LDL-c, low-density lipoprotein cholesterol; Glu, glucose; SA, stable angina; PLT, platelet.

### 3.2 Characteristics of Patients With CAD Based on GDF-15 Tertiles

We also analyzed the characteristics of patients with CAD stratified by GDF-15 tertiles (Table 2). Patients with the higher tertile of GDF-15 were more likely to have diabetes and hypertension, higher levels of inflammatory markers (WBC, Neu, and Mono) and lipids (high TC, TG, and low HDL-c), and a high Gensini score. No significant differences were observed among the three subgroups in terms of glucose, LDL-c, and PLT (*p* > 0.05). Regarding coagulation parameters, we observed significant differences among the three subgroups of GDF-15. Generally, PT and APTT were prolonged, and fibrinogen and D-dimer levels increased with increasing GDF-15 tertiles, while the activity of AT3 was significantly decreased.

**Table 2.** **Baseline characteristics of patients with CAD and different tertiles of GDF-15 levels**.

Characteristics	GDF-15 level			*p*-value
	Low (n = 195)	Medium (n = 196)	High (n = 201)	
	<999.35 pg/mL	999.35–1690.52 pg/mL	≥1690.52 pg/mL	
Age, years, median (IQR)	56.00 (51.00–62.00)^a^	64.00 (57.00–70.00)^b^	69.00 (63.00–75.00)^c^	<0.001
Diabetes, n, (%)	36 (18.46)^a^	55 (28.06)^b^	66 (32.84)^b^	<0.001
Hypertension, n, (%)	102 (52.31)^a^	121 (61.73)^b^	137 (68.16)^b^	0.005
Statin use, n (%)	47 (24.10)^a^	49 (25.00)^a^	52 (25.87)^a^	0.921
Aspirin use, n (%)	82 (42.05)^a^	77 (39.29)^a^	82 (40.80)^a^	0.856
Smoking, n, (%)	111 (56.92)^a^	109 (55.61)^a^	130 (64.68)^a^	0.138
PT (s)	10.6 (10.2–11.3)^a^	10.8 (10.2–11.6)^a^	11.4 (10.8–12.4)^b^	<0.001
APTT (s)	27.6 (25.1–29.7)^a^	27.7 (25.3–30.1)^a^	28.5 (26.2–31.8)^b^	0.003
TT (s)	17.9 (17.0–18.4)^a^	17.7 (16.9–18.5)^a^	17.3 (16.5–18.0)^b^	<0.001
FIB (g/L)	2.5 (2.1–3.0)^a^	2.7 (2.3–3.3)^b^	3.0 (2.5–3.8)^c^	<0.001
D-dimer (mg/L)	0.20 (0.14–0.40)^a^	0.30 (0.18–0.46)^b^	0.57 (0.31–1.27)^c^	<0.001
AT3 (%)	98.2 (91.2–104.9)^a^	93.1 (84.1–100.0)^b^	88.4 (78.9–99.9)^b^	<0.001
PLT	210.12 ± 61.35^a^	202.73 ± 63.73^a^	203.34 ± 82.33^a^	0.524
WBC (10^9^/L)	6.50 (5.38–7.55)^a^	6.70 (5.33–7.98)^a,b^	6.75 (5.54–8.77)^b^	0.043
Neu (10^9^/L)	3.90 (2.99–4.81)^a^	4.01 (3.30–5.30)^a^	4.39 (3.34–6.05)^b^	0.001
Lym (10^9^/L)	1.70 (1.33–2.12)^a^	1.64 (1.30–2.04)^b^	1.33 (1.03–1.88)^b^	<0.001
Mono (10^9^/L)	0.48 (0.41–0.61)^a^	0.54 (0.44–0.67)^b^	0.59 (0.45–0.72)^b^	<0.001
Glu (mmol/L)	4.91 (4.52–5.71)^a^	5.20 (4.56–6.98)^a,b^	5.29 (4.49–7.01)^b^	0.050
TC (mmol/L)	3.58 (3.10–4.24)^a^	3.84 (3.07–4.66)^a,b^	3.84 (3.21–4.68)^b^	0.026
TG (mmol/L)	1.21 (0.90–1.76)^a^	1.33 (0.95–1.81)^b^	1.56 (1.05–2.14)^b^	0.001
HDL-c (mmol/L)	0.93 (0.83–1.14)^a^	0.95 (0.79–1.11)^a^	0.88 (0.72–1.08)^b^	0.006
LDL-c (mmol/L)	2.11 (1.52–2.69)^a^	2.00 (1.41–2.78)^a^	2.00 (1.50–2.60)^b^	0.737
Gensini Score	16.50 (4.50–33.75)^a^	22.50 (8.00–55.00)^b^	32.00 (14.50–77.25)^c^	<0.001

Data are expressed as median (25th–75th percentile); Kruskal-Wallis test and one-way ANOVA were used for comparison. Different lowercase letters indicate significant differences between the groups (Dunn’s test or LSD test). PT, prothrombin time; APTT, activated partial thromboplastin time; TT, thrombin time; FIB, fibrinogen; AT3, antithrombin III; IQR, interquartile range.

### 3.3 Univariate Linear Regression Analyses

Univariate linear regression analyses were conducted to determine the correlations among GDF-15 levels, inflammatory markers, and blood lipids in all participants and subgroups. GDF-15 was positively correlated with age, leukocyte, neutrophil, monocyte count, PT, APTT, and D-dimer, and negatively correlated with lymphocyte count, TC, and AT3 in all patients with CAD and in the AMI subgroup (Tables 3,4). In contrast, in patients with SA, only age was positively correlated with GDF-15 levels (Table 5).

**Table 3.** **Univariate linear regression analyses in patients with CAD**.

Independent variables	Unstandardized coefficient (B)	95% CI for B	Standardized coefficient (β)	*p*-value
Age	65.590	49.639 to 81.542	0.315	<0.001
WBC	243.657	187.005 to 300.309	0.333	<0.001
Neu	294.139	236.025 to 352.254	0.384	<0.001
Lym	–699.972	–972.952 to 426.992	–0.206	<0.001
Mono	1361.550	754.810 to 1968.289	0.181	<0.001
PLT	–0.755	–3.270 to 1.761	–0.025	0.556
Glu	–0.185	–11.419 to 11.049	–0.001	0.974
TC	–181.127	–344.696 to –17.559	–0.093	0.030
TG	–26.504	–73.442 to 20.433	–0.047	0.268
HDL-c	–10.144	–47.787 to 27.490	–0.024	0.597
LDL-c	7.253	–192.378 to 206.885	0.003	0.943
PT	151.416	99.414 to 203.418	0.235	<0.001
APTT	47.264	25.066 to 69.462	0.178	<0.001
TT	–11.955	–38.776 to 14.866	–0.038	0.382
FIB	130.204	4.691 to 255.716	0.088	0.042
D-dimer	70.064	11.553 to 128.574	0.101	0.019
AT3	–3.301	–6.555 to –0.046	–0.095	0.047

Univariate linear regression analyses were employed to test the correlation between GDF-15 and each parameter in all patients with CAD.

**Table 4.** **Univariate linear regression analyses in patients with AMI**.

Independent variables	Unstandardized coefficient (B)	95% CI for B	Standardized coefficient (β)	*p*-value
Age	57.480	35.389 to 79.571	0.240	<0.001
WBC	252.800	185.770 to 319.829	0.340	<0.001
Neu	308.308	239.442 to 377.174	0.395	<0.001
Lym	–788.630	–1134.704 to –442.556	–0.214	<0.001
Mono	1171.101	453.298 to 1888.904	0.155	0.001
PLT	–0.731	–3.887 to 2.425	–0.022	0.649
Glu	39.726	–28.593 to 108.045	0.057	0.254
TC	–193.291	–417.650 to 31.067	–0.085	0.091
TG	–48.209	–137.355 to 40.937	–0.053	0.288
HDL-c	–601.281	–1497.877 to 295.316	–0.070	0.188
LDL-c	10.100	–245.250 to 265.451	0.004	0.938
PT	172.638	107.154 to 238.122	0.250	<0.001
APTT	46.476	20.580 to 72.373	0.177	<0.001
TT	–61.691	–199.128 to 75.746	–0.045	0.378
FIB	67.170	–80.111 to 214.452	0.046	0.370
D-dimer	151.982	58.323 to 245.642	0.160	0.002
AT3	–34.680	–51.718 to –17.642	–0.224	<0.001

Univariate linear regression analyses were employed to investigate the correlation between GDF-15 and each parameter among patients with AMI.

**Table 5.** **Univariate linear regression analyses in patients with SA**.

Independent variables	Unstandardized coefficient (B)	95% CI for B	Standardized coefficient (β)	*p*-value
Age	17.917	12.950 to 22.883	0.492	<0.001
WBC	19.294	–6.496 to 45.085	0.120	0.141
Neu	25.537	–0.778 to 51.852	0.155	0.057
Lym	–70.783	–156.050 to 14.483	–0.133	0.103
Mono	124.182	–176.464 to 424.828	0.067	0.416
PLT	–0.636	–1.376 to 0.103	–0.138	0.091
Glu	0.141	–1.513 to 1.795	0.014	0.866
TC	–2.677	–43.920 to 38.566	–0.011	0.898
TG	1.779	–6.536 to 10.094	0.035	0.673
HDL-c	0.616	–4.279 to 5.510	0.021	0.804
LDL-c	–5.128	–64.172 to 53.915	–0.014	0.864
PT	–7.427	–24.456 to 9.602	–0.070	0.390
APTT	–2.692	–14.815 to 9.432	–0.036	0.661
TT	–1.060	–5.132 to 3.012	–0.042	0.608
FIB	44.864	–30.448 to 120.176	0.096	0.241
D-dimer	–5.451	–17.128 to 6.225	–0.075	0.358
AT3	–0.403	–0.896 to 0.089	–0.142	0.108

Univariate linear regression analyses were employed to measure the correlation between GDF-15 and each parameter in patients with SA.

### 3.4 Multivariate Linear Regression Analyses

Multivariate linear regression analyses were conducted to determine the associations between GDF-15 levels, inflammatory markers, and blood lipids among all participants with CAD and subgroups of CAD. In patients with CAD, GDF-15 levels were positively associated with neutrophil count (β = 0.330, *p* < 0.001) and APTT (β = 0.109, *p* = 0.024) and inversely associated with AT3 (β = –0.113, *p* = 0.028) after adjusting for confounders (Table 6). After adjusting for confounders, GDF-15 levels were positively associated with monocyte count (β = 0.400, *p* < 0.001) and inversely associated with AT3 (β = –0.191, *p* = 0.036) in patients with SA (Table 7). In patients with AMI, GDF-15 levels were positively associated with APTT (β = 0.174, *p* = 0.002) and neutrophil count (β = 0.439, *p* < 0.001) and inversely associated with monocyte count (β = –0.159, *p* = 0.025) in adjusted multivariate regression model (Table 8).

**Table 6.** **Multivariate linear regression analyses conducted in all patients with CAD**.

Independent variables	Unstandardized coefficient (B)	95% CI for B	Standardized coefficient (β)	*p*
Age	54.677	36.511 to 72.843	0.293	<0.001
Neu	233.028	168.621 to 297.434	0.330	<0.001
APTT	50.316	6.594 to 94.039	0.109	0.024
AT3	–14.993	–28.379 to –1.606	–0.113	0.028

Multivariate linear regression analyses were employed to determine the associations among GDF-15 levels, inflammatory markers, and blood lipids among all participants with CAD. Analyses were adjusted for hypertension, diabetes, and smoking status.

**Table 7.** **Multivariate linear regression analyses in patients with SA**.

Independent variables	Unstandardized coefficient (B)	95% CI for B	Standardized coefficient (β)	*p*
Age	17.387	11.845 to 22.930	0.527	<0.001
Mono	623.158	371.734 to 874.581	0.400	<0.001
TG	9.072	2.567 to 15.576	0.247	0.007
AT3	–3.884	–7.507 to –0.260	–0.191	0.036

Multivariate linear regression analyses were employed to investigate the associations between GDF-15 levels, inflammatory markers, and blood lipids in all patients with SA. Analyses were adjusted for hypertension, diabetes, and smoking status.

**Table 8.** **Multivariate linear regression analyses in patients with AMI**.

Independent variables	Unstandardized coefficient (B)	95% CI for B	Standardized coefficient (β)	*p*
Age	49.890	26.182 to 73.597	0.234	<0.001
Neu	317.840	217.330 to 418.350	0.439	<0.001
Mono	–1007.947	–1885.992 to –129.903	–0.159	0.025
APTT	86.766	31.477 to 142.056	0.174	0.002

Multivariate linear regression analyses were used to assess the associations between GDF-15 levels, inflammatory markers, and blood lipids among all patients with AMI. Analyses were adjusted for hypertension, diabetes, and smoking status.

## 4. Discussion

This retrospective study analyzed data from 592 patients with CAD to investigate the relationship between circulating GDF-15 levels and coagulation parameters. The results showed a positive association between GDF-15 and APTT and and inverse association between GDF-15 and AT3 after controlling for potential confounders. To our best knowledge, this was the first study to investigate the relationship between GDF-15 and coagulation parameters among male patients with CAD.

GDF-15 is typically expressed at low levels in various organs, including the liver, lungs, and kidneys, but is upregulated in chronic diseases [27]. A substantial body of research indicates that elevated GDF-15 level is a strong and independent predictor of mortality and disease progression in patients with atherosclerosis and CAD, such as SA and ACS [28, 29, 30]. Importantly, CAD is an age-related condition associated with chronic inflammation. Inflammation was shown to affect the development of atherosclerosis, including its initiation, progression, rupture and thrombosis [31]. Angiogenesis plays a significant role in the progression of atherosclerotic plaques and complications [32, 33]. The local inflammatory response of atherosclerotic plaques promotes angiogenesis by activating endothelial cells, releasing chemokines, cytokines, growth factors, lipid mediators, and proteases and increasing endothelial metabolism. Angiogenesis allows the extravasation of plasma components, leading to thromboembolic events [34]. Plaque angiogenesis and intraplaque hemorrhage are the key factors leading to unstable lesions [35]. De Jager *et al*. [12] and Bonaterra *et al*. [13] suggested that GDF-15 may modulate monocyte and macrophage activation, contributing to a prothrombotic state via cytokine-driven endothelial dysfunction and tissue factor expression. Moreover, Dong *et al*. [36] revealed that GDF-15 can phosphorylate Src and its downstream pathways to induce the pro-angiogenic effects. These findings imply the regulatory role of GDF-15 in angiogenesis and cell proliferation in atherosclerosis [36]. Based on these findings, GDF-15 may affect coagulation pathways by regulating inflammation and angiogenesis in CAD, especially in ACS.

GDF-15 increases the risk of atherosclerosis through multiple mechanisms. For instance, it promotes CCR2-mediated macrophage chemotaxis toward atherosclerotic plaques and interleukin-6-dependent inflammatory responses [12, 13]. In this study, the ACS and SA groups exhibited significantly higher levels of inflammatory markers (WBC, Neu, and Mono), glucose, and lipids (TG) and lower Lym count compared to controls (*p* < 0.01). These differences in inflammatory markers levels may reflect GDF-15 upregulation in response to inflammatory or stress stimuli [37]. Additionally, elevated serum levels of GDF-15 were found to be correlated with abnormal lipid profiles, aligning with the findings of previous studies linking GDF-15 to dyslipidemia [29, 38]. In this study, the concentration of TC in the control group was higher than that in SA and AMI. An explanation for this may be the appropriate levels and criteria for judging elevated LDL-c for different groups of people with varying atherosclerotic cardiovascular disease risks, as well as the LDL-c levels at which to initiate lipid-lowering drug treatment and the treatment targets for LDL-c, are all different [39, 40]. Consistently, our study confirmed higher levels of GDF-15 in patients with CAD than in controls. Activated platelets exacerbated the development of atherosclerosis by releasing chemokines, which dominate the inflammatory response in ACS [36]. There was no difference in terms platelet count among SA, AMI, and normal coronary groups, or between thrombotic and non-thrombotic myocardial infarction [41, 42]. In this study, platelet count was lower in patients with SA and AMI compared to controls. This might be due to the fact that the antiplatelet therapy was more stringent for patients with SA and AMI. In addition, we only analyzed the use of aspirin but omitted other antiplatelet such as ticagrelor and clopidogrel or anticoagulant agents due to the lack of data. In a previous study, circulating GDF-15 was positively and non-linearly associated with the prevalence of hypertension [43]. GDF-15 was reported to be regulated in diabetes and control insulin sensitivity in mice via the glial-cell-line-derived neurotrophic factor (GDNF) family receptor α-like receptor [44]. To analyze the relationship between GDF-15 and coagulation parameters, patients with CAD were stratified by GDF-15 tertile. Consistent with previous reports, higher tertiles of GDF-15 were associated with older age, diabetes, and hypertension, which are established risk factors for complications and mortality in those with CAD [45]. This study is the first to report the associations between GDF-15 and coagulation parameters. Matusik *et al*. [46] reported that GDF-15 is positively correlated with endogenous thrombin potential and clot lysis time. Fibrinogen is a key component of atherogenesis [47], contributing to thrombosis, inflammation, and blood viscosity. Lippi *et al*. [48] reported that GDF-15 is independently correlated with platelet function and fibrinogen levels in healthy adults. These findings explain the positive correlation between GDF-15 and fibrinogen in univariate linear regression analysis. After adjusting for confounders, multivariate linear regression analyses indicated that GDF-15 levels were positively associated with APTT and inversely associated with AT3. Antithrombin is a critical serine protease inhibitor whose heparin-bound form mediates anticoagulation [49]. ACS involves acute coronary occlusion, impairing the balance between coagulation and anticoagulation, suggesting that GDF-15 may offer additional insights into the coagulation status in ACS.

In addition to its associations with inflammation and stress, elevated levels of GDF-15 have been independently linked to an increased risk of stroke, systemic embolic events, and major bleeding [50, 51, 52]. Notably, Siegbahn *et al*. [53] found that high concentrations of GDF-15 significantly predict major bleeding in patients with atrial fibrillation receiving anticoagulants. Furthermore, Matusik *et al*. [46] reported that elevated levels of GDF-15 independently predict impaired fibrin clot lysis in patients with atrial fibrillation, possibly due to its association with prothrombotic alterations. Together, these studies highlight the importance of GDF-15 in coagulation.

Several limitations of this study should be acknowledged. Firstly, there was a lack of data regarding the relationship between GDF-15 levels and clinical outcomes, including survival and major adverse cardiovascular events (MACE). Secondly, the study exclusively enrolled male patients with CAD. Although this approach was adopted to control for gender-specific confounding factors, significant gender differences exist regarding the prevalence of CAD. Thirdly, as a case-control study with a relatively small sample size, the present study can only demonstrate an association between GDF-15 and coagulation parameters, but could not establish a causal relationship. Future studies with larger sample sizes or longitudinal designs are warranted to elucidate this relationship. Finally, this study only assessed the use of aspirin and ignored other antiplatelet (e.g., ticagrelor, clopidogrel) or anticoagulant agents (e.g., NOACs, warfarin), and future study is required for further investigation of this relationship.

## 5. Conclusions

In summary, this study showed significant associations between elevated serum levels of GDF-15 and multiple coagulation parameters, particularly APTT and AT3. These findings suggest that GDF-15 can serve as a compensatory marker for coagulation instability. These results underscore the potential clinical application of GDF-15 as a novel biomarker for assessing the coagulation status in male patients with CAD, especially in the ACS subgroup.

## Data Availability

All data generated or analyzed during this study are included in this article. Further studies should be directed toward the corresponding authors.

## Abbreviations

WBC, white blood cell; Neu, neutrophil, Lym, lymphocyte; Mono, monocyte; Glu, glucose; TC, total cholesterol; TG, triglycerides; HDL-c, high-density lipoprotein cholesterol; LDL-c, low-density lipoprotein cholesterol; GDF-15, growth differentiation factor-15; PT, prothrombin time; APTT, activated partial thromboplastin time; TT, thrombin time; FIB, fibrinogen; AT3, antithrombin III; PLT, platelet; CAD, coronary artery disease; SA, stable angina; ACS, acute coronary syndrome; AMI, acute myocardial infarction; GNDF, glial-cell-line-derived neurotrophic factor.
